# Neutrophil Extracellular Traps Caused by Gut Leakage Trigger the Autoimmune Response in Nonobese Diabetic Mice

**DOI:** 10.3389/fimmu.2021.711423

**Published:** 2022-01-17

**Authors:** Qi You, Yiming Shen, Yiling Wu, Yuyan Li, Chang Liu, Fengjie Huang, Harvest F. Gu, Jie Wu

**Affiliations:** ^1^ College of Life Science and Technology, China Pharmaceutical University, Nanjing, China; ^2^ State Key Laboratory of Natural Medicines, China Pharmaceutical University, Nanjing, China; ^3^ Center for Pathophysiology, School of Basic Medicine and Clinical Pharmacy, China Pharmaceutical University, Nanjing, China

**Keywords:** autoimmunity, gut barrier function, neutrophil extracellular traps, peptidyl arginine deiminase type 4, type 1 diabetes

## Abstract

Increased formation of neutrophil extracellular traps (NETs) is associated with gut leakage in type 1 diabetes (T1D). To explore the mechanism of how enteropathy exacerbated by NETs triggers pancreatic autoimmunity in T1D, we carried out a correlation analysis for NET formation with gut barrier functions and autoimmunity in nonobese diabetic (NOD) mice. Inducing chronic colitis or knocking out of peptidyl arginine deiminase type 4 (PAD4) in NOD mice were used to further study the effect of NET formation on the progression of T1D. Microbial alterations in Deferribacteres and Proteobacteria, along with the loss of gut barrier function, were found to be associated with increased endotoxin and abnormal formation of NETs in NOD mice. Both DSS-induced colitis and knockout of PAD4 in NOD mice indicated that PAD4-dependent NET formation was involved in the aggravation of gut barrier dysfunction, the production of autoantibodies, and the activation of enteric autoimmune T cells, which then migrated to pancreatic lymph nodes (PLNs) and caused self-damage. The current study thus provides evidence that PAD4-dependent NET formation is engaged in leaky gut triggering pancreatic autoimmunity and suggests that either degradation of NETs or inhibition of NET formation may be helpful for innovative therapeutic interventions in T1D.

## Introduction

Type 1 diabetes (T1D) is a chronic autoimmune disease characterized by insulin-producing pancreatic beta cells destroyed by autoreactive T cells (1, 2). Adequate evidence has been provided to confirm the leaky gut-affecting pancreas pathogenic theory. For instance, the development of clinical diabetes in patients and preclinical models (diabetes-prone rats) of T1D is often preceded by increased intestinal permeability (1, 2). It has been proposed that the loss of gut barrier function caused by enteric infections (3), commensal microbiota dysbiosis (4, 5), dietary antigen exposure, and local inflammation (6) could lead to the uncontrolled invasion of bacterial components, followed by the generation of islet-reactive CD4- or CD8-positive T cells (7) in gut-associated lymphoid tissue (GALT), and these cells possess PLN homing abilities and ultimately induce insulitis (8–10). However, in this process, the transition from innate immunity to adaptive immunity after losing gut barrier integrity has rarely been mentioned.

Neutrophils are essential innate immune cells in the recognition and elimination of invasive pathogens (11), and their contribution to the pathogenesis of T1D was demonstrated in NOD mice, in which neutrophils infiltrated the pancreas and could form NETs (12). In line with this finding, in T1D patients, a reduced circulating neutrophil number was accompanied by the rise of neutrophils or NETs in their pancreas (13, 14). Our previous clinical study suggested that NET formation is increased in new-onset T1D patients compared with healthy controls (15). Parackova et al. have recently indicated that NET-induced dendritic cell activation led to Th1 polarization in T1D patients (16). As a special way of capturing invasive bacteria, NETs contain abundant cytosolic proteins, especially those with post-translational modifications, such as PAD4 that drives citrullination, which is an important potential source of self-antigens to promote the loss of immune tolerance and autoimmunity (17) and increase the generation of antineutrophil cytoplasmic antibodies (ANCAs) (18) and anti-cyclic citrullinated peptide antibodies (ACPAs) (19). Masses of self-DNA sprayed in this process were proven to be capable of activating plasmacytoid dendritic cells (pDCs) or monocytes through cGAS-STING and triggering proinflammatory cytokines to drive autoimmune pathology in T1D patients (20). In addition, B cells activated by self-DNA produce an anti-dsDNA antibody to trigger an autoimmune response (21).

We recently reported that degradation of NETs in the gut of NOD mice by nuclease production due to recombinant *Lactococcus lactis* could protect against T1D development (22). In the current study, we aimed to investigate whether NETs participation in the T1D progression is triggered by gut leakage. First, we verified the correlation between abnormal NET formation and gut leakage in preclinical models of autoimmune T1D. Then we investigated whether aggravated gut leakage induced by 2% dextran sulfate sodium (DSS) could increase NET formation and accelerate T1D occurrence in NOD mice accordingly. Finally, we examined whether decreased NET formation and improved gut barrier function could alleviate T1D progression in PAD4 knockout mice. Thereby, data from the current study may reveal that NET mediator roles in the progression of T1D are triggered by intestinal barrier breakage and associated with both cell-mediated immunity and humoral immunity.

## Methods and Materials

### Animals

Both NOD/LtJ and BALB/c mice were female and purchased from Huafukang Bioscience Co., Inc. (Beijing, China). PAD4-/- mice with the NOD/LtJ background were purchased from Animal Model Research Center of Nanjing University (Nanjing, China). All mice were maintained under specific pathogen-free conditions in Animal Laboratory Center of China Pharmaceutical University (CPU, Nanjing, China). Animal care and all experimental methods were performed following the relevant guidelines and regulations and approved by the local ethics committee in CPU.

### Induction of Chronic DSS Colitis

A solution of 2% (wt/vol) DSS (molecular weight: 36,000–50,000 Da; MP Biomedicals, Solon, USA) was orally administered to 4-week-old NOD/LtJ mice in drinking water for three cycles of 7 days at intervals of 2 weeks before the mice were at 13 weeks old and an additional three cycles of 7 days at intervals of 1 week afterward.

### Neutrophil Isolation and NETs Assay

Spleen neutrophils were isolated with Percoll (Solarbio, Beijing, China) gradients as described (23). NETs assay was performed as protocols published (24). Neutrophils were resuspended in RPMI-1640 containing 5-10% FBS and plated at 5×10^5^ cells per well in 24-well plates (Thermo Fisher, Massachusetts, US). After incubation for 0.5-1 h, the cells were stimulated with PMA (8 μM, MedChemExpress, New Jersey, USA), ionomycin (10 μM, MedChemExpress, New Jersey, USA) or fecal microbiota (2×10^7^ CFU), and the neutrophils were incubated for an additional 3 h to form NETs. Cells were stained with SYTOX green (1:1000, KeyGEN BioTech, Nanjing, China) for NETs quantification or, in some experiments, fixed in 2% paraformaldehyde, permeabilized, blocked, and stained with anti-H3cit (1:1000, Abcam, cat. no. ab5103) and anti-rabbit secondary antibodies (1:200, Servicebio, Wuhan, China). Images were acquired on an Axiovert 200 M wide-field fluorescence microscope (Nikon) with a coupled camera. The percentages of H3cit high cells and NETs were determined from five or six nonoverlapping fields per well, and the average was taken from 2-3 biological repetitions in every experiment.

### Mononuclear Cell Isolation and Co-Culture With NETs

First, spleen mononuclear cells were isolated using Percoll (Solarbio, Beijing, China) gradients as described (25). After stimulating 5×10^5^ neutrophils with or without ionomycin *in vitro*, the culture medium was carefully removed, the NETs/neutrophils were washed slightly 2-3 times by RPMI-1640 (10% FBS) and resuspended in 300 μL RPMI-1640 containing 5-10% FBS. NETs/neutrophils were then added to wells containing 5×10^6^ mononuclear cells in 500 μL culture medium and co-cultured for 7 days, with medium added every two days.

### Min6 Cell Culture and *In Vitro* Lymphocyte Cell Migration Assay

Min6 beta cells (CL-0674, Procell Life Science&Technology Co., Ltd. Wuhan, China) were cultured with RPMI-1640 (10% FBS, 50 mM β-mercaptoethanol) for 24 h (authentication and no mycoplasma contamination reports were provided by the supplier), and Min6 cell-containing medium was transferred to the bottom of 24-well Boyden chambers (NEST, Wuxi, China) for an additional 12 h of culture. A total of 1×10^5^ lymphocyte cells was seeded onto the porous permeable membrane in the upper chambers. After 12-14 h, the migrated cells in the bottom chamber were collected and counted.

### Diabetes Incidence

Diabetes was weekly monitored by measuring blood glucose levels with a Boshilong blood glucose meter (Houmeide Biotechnology, Taipei, China). After two consecutive blood glucose measurements ≥13.8 mmol/L, the mice were then considered to be diabetic.

### 16S rRNA Microbiota Analysis

Total genomic DNA from samples was extracted by using the CTAB/SDS (Sangon Biotech, Shanghai, China) method. 16S rRNA genes were amplified using barcoded sample-specific primers: 16S V4-V5: 515F-907R. All PCRs were carried out in 30 μL reactions with 15 μL of Phusion^®^ High-Fidelity PCR Master Mix (New England Biolabs (Beijing) LTD, Beijing, China), 0.2 μM forward and reverse primers, and approximately 10 ng template DNA. Thermal cycling consisted of initial denaturation at 98°C for 1 min, followed by 30 cycles of denaturation at 98°C for 10 s, annealing at 50°C for 30 s, and elongation at 72°C for 60 s. Finally, samples were held at 72°C for 5 min. Amplicons were loaded onto a 2% agarose gel, and samples with bright main strips between 400-450 bp were chosen for further experiments. PCR products were mixed in equidensity ratios. Then, the mixed PCR products were purified with a Gene JET Gel Extraction Kit (Thermo Scientific, Waltham, USA). Sequencing libraries were generated using the NEB Next ^®^ UltraTM DNA Library Prep Kit for Illumina (New England Biolabs (Beijing) LTD, Beijing, China) following the manufacturer’s recommendations, and index codes were added. The library quality was assessed on the Qubit@ 2.0 Fluorometer (Thermo Scientific, Waltham, USA) and Agilent Bioanalyzer 2100 system (Agilent Technologies, Beijing, China). Finally, the library was sequenced on an Illumina MiSeq platform (Illumina, San Diego, USA), and 250 bp/300 bp paired-end reads were generated. Paired-end reads were assigned to each sample according to the unique barcodes, and sequence analysis was performed by the UPARSE software package using the UPARSE-OTU and UPARSE-OTU ref algorithms.

### Histopathological Analysis and Immunofluorescence

The pancreas and colon were fixed in 4% paraformaldehyde and embedded in paraffin (Servicebo, Wuhan, China). To assess histopathological signs of diabetes, consecutive sections (4 μm) were then stained with hematoxylinand eosin (H&E, Sangon Biotech, Shanghai, China) and scored for insulitis, in which 0 stood for no infiltration, 1 stood for periinsulitis, 2 stood for infiltration covering approximately half of the islet, 3 stood for infiltration covering approximately 75% of the islet and 4 stood for full insulitis. For colon sections, after H&E staining, three independent parameters were determined: the depth of injury (scores 0, 1, 2 and 3 for none, mucosal, mucosal/submucosal and transmural, respectively), muscle layer thickness, and the ratio of villus height/crypt depth. Sections were also stained with PAD4 (Proteintech, Wuhan, China), Ly6G (Servicebio, Wuhan, China), H3cit (Abcam) and insulin (Servicebio, Wuhan, China) and subsequent fluorescein-labeled secondary antibodies to display NET formation or insulin production. Histological staining of mucins and calculation of mucus thickness were conducted according to previous descriptions (26).

### NET-Associated Biomarkers and T1D Autoantibody Detection

Serum samples and pancreas tissue homogenates were all stored at -80°C and thawed at 4°C for 20 min before testing. Circulating NE, MPO, PAD4, ACPA, dsDNA-Ab, IAA, ZnT8A, IA2A and GADA were measured individually by commercial enzyme-linked immunosorbent assay (ELISA) kits, according to the manufacturer’s protocols (Tongwei Biotech, Shanghai, China). Absorbance was measured using a multifunction microporous plate detector (Spark, Tecan Group Ltd, Männedorf, Switzerland). For the detection of ANCAs in serum, first, NET formation operated just like the methods “Neutrophil isolation and NETs assay” showed. Then, the supernatant was discharged carefully by slow suction and micrococcal nuclease (1 U/mL, Solarbio, Beijing, China) was added to digest NETs at 37°C for 20 min followed by 5 mM EDTA to stop the nuclease activity. The supernatant was collected and centrifuged to eliminate cell debris. The total cytoplasmic content was diluted 1:300 in buffer (0.1 M Na2CO3, 0.1 M NaHCO3, pH=9.6) and incubated at 4°C overnight in 96-well plates. After blocking with 5% BSA (37°C, 2 h), the serum samples were diluted 1:200 in 5% BSA and incubated at 37°C for 2 h. After washing with PBST (PBS containing 0.5% Tween 20), HRP-conjugated goat anti-mouse IgG (1:3000, Servicebio, Wuhan, China) was added and incubated at 37°C for 1 h. TMB was added to all wells (Beyotime Biotechnology, Shanghai, China), and the samples were incubated in the dark for 30 min at 37°C, then 2 M H2SO4 was used to stop the reaction. The absorbance at 450 nm was measured using a multifunction microporous plate detector (Spark, Tecan Group Ltd, Männedorf, Switzerland). To assess cf-DNA levels *in vivo*, the PICOGREEN^®^, Quant-iT™ dsDNA Assay Kit (Thermo Fisher, Massachusetts, US) was used according to the manufacturer’s protocols. To detect circulating H3cit levels, anti-H3cit (1:1000, Abcam) and anti-H3 (1:3000, Proteintech, Wuhan, China) were diluted in buffer and followed the routines mentioned above.

### LPS Detection in Serum

Serum samples were all stored at -80°C and thawed at 4°C for 20 min before testing. LPS was measured individually by commercial ELISA kits, according to the manufacturer’s protocols (Tongwei Biotech, Shanghai, China). Absorbance was measured using a multifunction microporous plate detector (Spark, Tecan Group Ltd, Männedorf, Switzerland).

### Cross-Reaction of Insulin Autoantibody (IAA) With NETs Associated Auto-Antigens

Lysed NETs (1:300) or insulin (1:200, Yuanye, Shanghai, China, 1 mg/ml) was diluted in buffer (0.1 M Na2CO3, 0.1 M NaHCO3, pH=9.6) and incubated at 4°C overnight in 96-well plates. After blocking these two plates with 5% BSA (37°C, 2 h), the serum samples were diluted 1:200 in 5% BSA and incubated with NET protein at 37°C for 2 h. Another uncoated plate was also blocked with 5% BSA (37°C, 2 h) and then incubated with the same diluted samples at the same time. Next, all samples from NET-coated plates or uncoated plates were collected and added to insulin-coated plates, followed by 2 h of incubation at 37°C. After subsequent routines, the differences in absorbance were compared between pretreated subjects and untreated subjects of the same sample. The cross-reaction ratio =(OD _(uncoated+insulin)_ - OD _(NET protein+insulin)_)/OD _(uncoated+insulin)_

### Western Blotting

Pancreatic samples were snap-frozen and homogenized in RIPA buffer (Sangon Biotech, Shanghai, China) on ice. After centrifugation at 12000 rpm for 10 min at 4°C, the protein content of the supernatant was mixed with loading buffer. An equal amount of protein per sample was resolved on gradient gels (15% Tris-glycine gels) and electroblotted on PVDF membranes, which were then incubated with primary antibodies (rabbit polyclonal anti-H3cit, Abcam; rabbit polyclonal anti-GAPDH, Servicebo) at 4°C overnight and subsequently with appropriate HRP-conjugated secondary antibodies (Servicebo, Wuhan, China) for 1 h at room temperature. The blots were developed with enhanced chemiluminescence substrate (Sangon Biotech, Shanghai, China). Blots were quantified using ImageJ software.

### *In Vivo* Permeability Assay

Intestinal permeability was determined by FITC-dextran assay as previously described (8). First, 20 mL/kg of body weight PBS containing 20 mg/mL FITC-conjugated dextran (FITC-dextran; molecular mass, 4.4 kDa; FD4, Sigma-Aldrich) was administered to each mouse by oral gavage. Three hours later, blood was collected in anticoagulant tubes and centrifuged to obtain the plasma (4000 rpm, 5 min at 4°C). Then, 50 ml of plasma was added to a 96-well microplate. The concentration of fluorescein was determined by a multifunction microporous plate detector (Spark, Tecan Group Ltd, Männedorf, Switzerland) with an excitation wavelength of 485 nm and an emission wavelength of 530 nm using serially diluted samples of the FITC-dextran marker as the standard.

### Oral Glucose Tolerance Test

Nondiabetic mice in each group (n=8) were selected for the oral glucose tolerance test (OGTT). After 12 h of fasting overnight, the basal blood glucose was detected, followed by oral administration of glucose solution (2g/kg). Then, the blood glucose was monitored at the following time points: 15 min, 30 min, 60 min and 90 min.

### Flow Cytometry

Analysis Single-cell suspensions from pancreatic lymph nodes (PLNs), mesenteric lymph nodes (MLNs), spleens and cultured cells were resuspended in staining buffer containing PBS and 1% FBS, stained with monoclonal antibodies against surface markers, fixed and permeabilized using the BD Cytofix/Cytoperm kit, and finally stained for intracellular transcription factors. The following antibodies were used: FITC anti-mouse CD4 (RM4-5; BD), APC anti-mouse CD25 (PC61; BioLegend), PE-cy7 anti-mouse ROR-γt (B2D; Invitrogen), APC anti-mouse T-bet (4B10; BioLegend), APC anti-mouse GATA-3 (16E10A2; BioLegend), Alexa Fluor 647 anti-mouse FoxP3 (MF23; BD), and APC anti-mouse α4β7 (DATK32; Proteintech).

### RNA Isolation and RT-qPCR

Immediately after the mice were sacrificed, the colon and pancreas were cut into pieces and placed in 500 μL RNA storage preserving fluid (Tiangen Biotech Beijing CO., LTD), followed by storage at -80°C. After homogenization in 500 μL TRIzol reagent (Sangon Biotech, Shanghai, China), the RNA was extracted by adding 100 μL of chloroform and precipitating the aqueous phase with 200 μL of isopropanol. cDNA was synthesized using HiFi Script cDNA Synthesis Kit (Sangon Biotech, Shanghai, China). RT-qPCR analyses were conducted to quantitate the relative mRNA expression using Ultra SYBR Mixture (Sangon Biotech, Shanghai, China). The primer sequences are shown in the **Supplemental Table 1**. Gene expression was normalized using GAPDH as the reference gene. To analyze the relative fold change, we employed the 2^−△△CT^ method.

### Statistical Analysis

SPSS 22.0 software (SPSS, Chicago, USA) was used for statistical analysis. The statistical significance of differences between two or more samples was calculated by an unpaired two-tailed Student’s t test or one ANOVA test. Correlations were analyzed by the Spearman correlation test. The cumulative diabetes incidence was calculated using Kaplan–Meier estimation, whereas the statistical significance was evaluated by the log-rank test. All data are presented as means ± SEM, and P<0.05 was used to indicate a statistically significant difference.

## Results

### Abnormal NET Formation Correlated With Loss of the Gut Barrier and Alterations in the Gut Microbiota in NOD Mice Before T1D Onset

**Figure 1:**
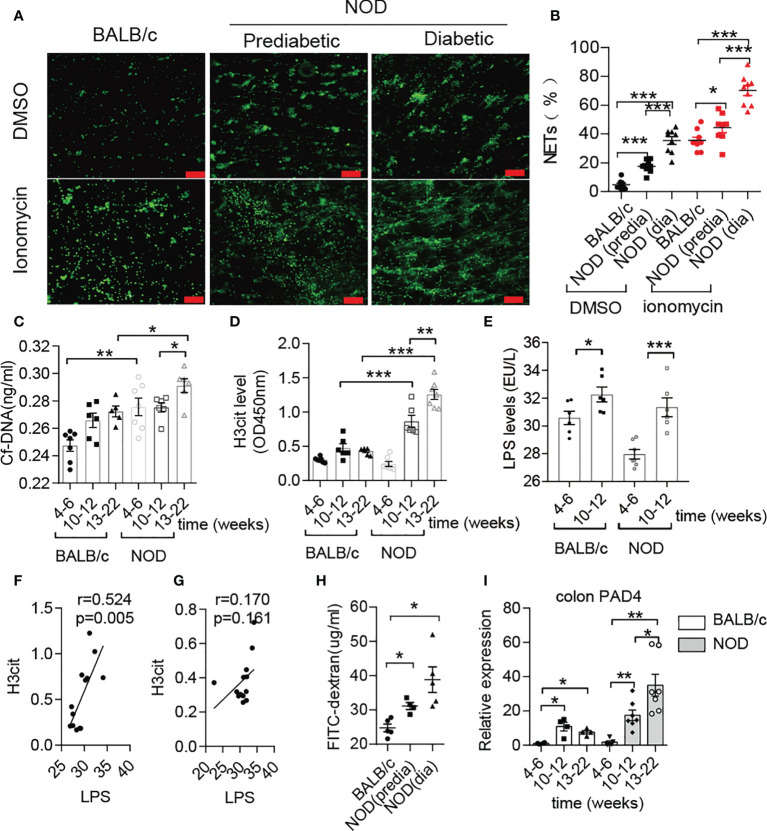
Abnormal NET formation correlated with increased LPS levels in NOD mice. **(A, B)** NET formation ability in NOD mice *vs.* BALB/c mice (n= 4). NET quantification was conducted twice for each mouse. Circulating levels of cf-DNA **(C)**, H3cit **(D)** and LPS **(E)** in NOD mice *vs.* BALB/c mice at different ages (n= 5-7). Spearman correlation analysis of H3cit **(F, G)** with LPS in NOD mice (n= 13) compared with BALB/c mice (n= 13). **(H)** FITC-dextran *in vivo* permeability assay in female BALB/c and NOD mice (n= 5). **(I)** RT-qPCR analysis of PAD4 in colons from NOD (n= 7, respectively, at different ages) and BALB/c mice (n= 4, respectively, at different ages). The scale bars indicate 100 μm **(A)**. Data are shown as the mean ± SEM. *P < 0.05, **P < 0.01, and ***P < 0.001.

Previous studies have mentioned that NOD mice exhibit prominently increased gut permeability compared to BALB/c mice at approximately 10-12 weeks old (8), and invasive microbes are indispensable for islet-reactive T cell action (27). However, little is known about the microbial changes at this time. Hence, we first conducted microbial analysis in NOD mice at four different stages (**Supplementary Figure 1A**). Interestingly, the abundance of Deferribacteres or Proteobacteria in NOD mice after 12 weeks was increased sharply when compared with mice before 12 weeks (**Supplementary Figure 1B**), and principal coordinates analysis (PCOA) exhibited the significant difference between the two groups (**Supplementary Figure 1C**). Moreover, we discovered that two gram-negative bacterial families, Desulfovibrionaceae and Deferribacteraceae, displayed significant augmentation, while the other two families, Muribaculaceae and Lactobacillaceae, which are short-chain fatty acid producers, were markedly reduced in NOD mice after 12 weeks (**Supplementary Figures 1D, E**). The increased abundance of Desulfovibrionaceae, an essential endotoxin producer, was in line with the increased endotoxin/lipopolysaccharide (LPS) level in the serum of NOD mice after 12 weeks of age (**Figure 1E**), which indicated a high risk of subsequently abnormal neutrophil-forming NETs in NOD mice. Then, an ionomycin stimulation experiment confirmed this finding and showed that neutrophils in NOD mice were easier to form NETs *in vitro* when compared with BALB/c mice, and diabetic NOD mice released more NETs compared to prediabetic mice (**Figures 1A, B**). In line with this, the circulating levels of NET biomarkers, such as cf-DNA and H3cit in NOD mice exhibited obvious differences from BALB/c. The serum concentrations of cf-DNA (**Figure 1C**) were higher in NOD mice at all times, and highly significant increases in H3cit and LPS was observed in NOD mice at 10-12 weeks of age compared with 4-6 weeks of age (**Figures 1D, E**). More importantly, the strong correlation between LPS and H3cit only in NOD mice (**Figure 1F**) but not BALB/c mice (**Figure 1G**) suggested that the loss of gut barrier function and the invasion of booming gram-negative bacteria led to the abnormal generation of NETs before T1D onset, which could be a potential pathogenic factor.

In addition to verifying increased intestinal permeability (**Figure 1H**), obvious histological lesions, higher gut damage scores and lower villus height/crypt depth ratios located in the colon of NOD mice after 10-12 weeks of age (**Supplementary Figures 2A, B**), we further observed the increased inflammatory cytokines IL-1β, IL-17 and TNF-α in colons of NOD mice (**Supplementary Figure 2C**). In contrast to BALB/c mice, continuously increased mRNA expression of PAD4 was detected in the NOD colon at different weeks (**Figure 1I**). Taken together, these results demonstrated that NET formation correlated with gut leakage and alterations in the gut microbiota in NOD mice.

### NETs Caused Cellular Immunity and Humoral Immunity in NOD Mice

**Figure 2:**
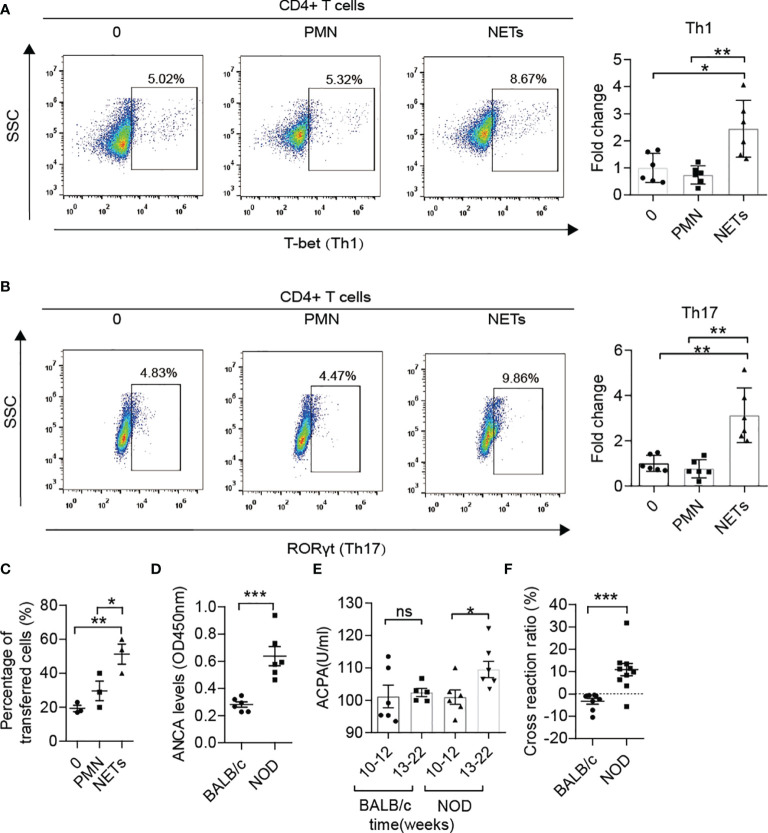
The roles of NETs in the autoimmunity of NOD mice. **(A, B)** NETs triggered the proliferation of memory Th1 and Th17 cells from spleen mononuclear cells in diabetic NOD mice *in vitro* (n= 6). **(C)** The Transwell system showed the migration capacity of lymphocyte cells from the MLN in diabetic NOD mice (n= 3). **(D)** Increased ANCA was detected in NOD mice in comparison with BALB/c mice after 12 weeks of age (n= 6). Serum levels of ACPA **(E)** in NOD mice *vs.* BALB/c mice at different ages (n= 6). **(F)** Cross-reaction of IAA with NET-associated autoantigens in NOD mice (n= 10) but not BALB/c mice (n= 8) after 12 weeks of age. Data are shown as the mean ± SEM. ^ns^P > 0.05, *P < 0.05, **P < 0.01, and ***P < 0.001.

Increased Th1 and Th17 and decreased Treg were observed in MLNs of NOD mice compared with BALB/c mice after 12 weeks of age (**Supplementary Figures 2D, E**) as similar to the previous report (8). The latest clinical research on T1D patients has demonstrated that NETs can polarize naïve T cells to Th1 cells by activating pDCs (16). In addition, numerous studies on autoimmune diseases have also shown the role of NETs in inducing immune dysfunction (17). However, no clues have been found to test this process in NOD mice thus far. Therefore, we isolated neutrophils from diabetic NOD mice and stimulated them with ionomycin to form NETs *in vitro*, followed by coculture with autologous spleen mononuclear cells. The proliferation of memory Th1 and Th17 cells indicated that NETs in NOD mice possess the capacity to activate proinflammatory T cells, which could trigger beta cells destruction and autoimmune diabetes (**Figures 2A, B**). We then tested the migration capacity of lymphocytes from the MLN in NOD mice. Neutrophils from the spleen were stimulated to form NETs *in vitro* and then cocultured with autologous lymphocytes from MLNs. The results of the transwell migration assay showed that more lymphocytes could migrate to the insulinoma cell line MIN6 *in vitro* (**Figure 2C**). Taken together, these findings indicated that enteric lymphocytes could be activated by NETs and possessed a stronger response to beta cells.

As reported in other autoimmune diseases, such as arthritis, lupus and vasculitis, ANCAs, ACPAs or anti-dsDNA antibodies play critical roles in self-destruction. ANCA levels in serum were also elevated in NOD mice after 12 weeks of age (**Figure 2D**) compared with BALB/c mice of the same age. Moreover, ACPA was sharply increased at disease onset in NOD mice but not in BALB/c mice (**Figure 2E**). To explore whether the components of NETs and insulin shared common epitopes, the cross-reaction was performed, and the results showed that NET-associated autoantibodies could indeed cross-react with insulin at a ratio of 10-20% (**Figure 2F**). Pretreatment with the components of NETs led to a significantly lower serum level of IAA in NOD mice but not in BALB/c mice after 12 weeks of age.

### DSS Triggered Increased NET Formation and Accelerated the Course of T1D

**Figure 3:**
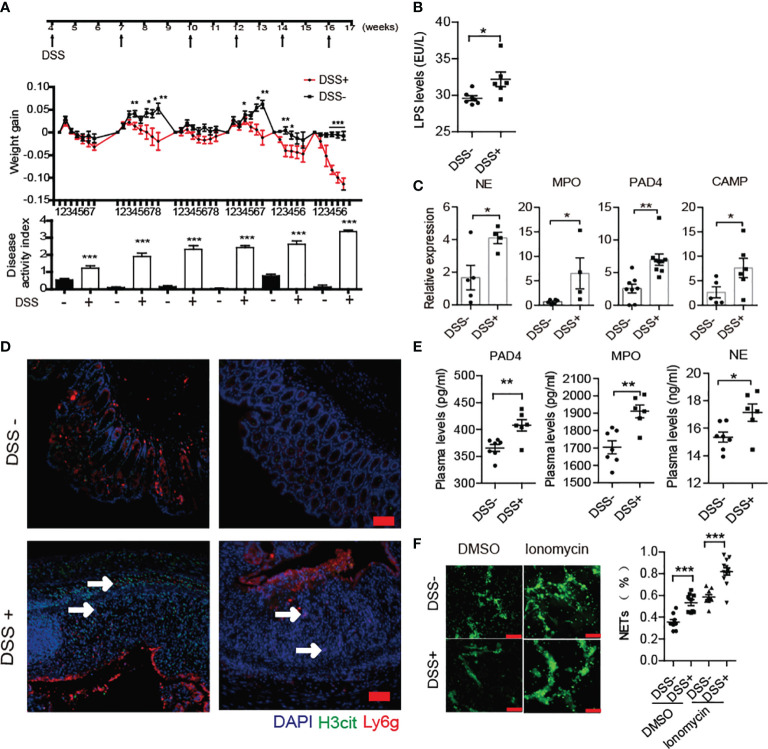
DSS treatment triggered increased NET formation in NOD mice. **(A)** Six administrations of DSS induced chronic colitis in control (n= 14) and DSS-induced colitis NOD mice (n= 19). **(B)** Increased circulating LPS levels in DSS- induced colitis NOD mice (n= 6). **(C)** Elevated expression of NE, MPO, CAMP and PAD4 were found in colon homogenates of DSS-induced colitis NOD mice *vs.* control mice. **(D)** Immunofluorescent staining of Ly6G, H3cit and PAD4 in colon sections of DSS-induced colitis NOD *vs.* control mice. **(E)** Increased circulating NET biomarker levels in DSS-induced colitis NOD (n= 6) compared with the control mice (n= 7). **(F)** DSS administration enhanced neutrophils’ capability of forming NETs *in vitro* (thrice NET quantification per mouse, n= 3). The scale bars indicate 50 μm **(D)** and 100 μm **(F)**. Data are shown as the mean ± SEM. *P < 0.05, **P < 0.01, and ***P < 0.001.

A previous report has shown that DSS-induced chronic colitis could trigger 60% BDC2.5XNOD mice developing T1D (8). In the current study, we performed similar methods for inducing loss of the gut barrier integrity through six administrations of DSS from 4 weeks old in NOD/LtJ to explore whether increased NET formation plays crosstalk roles between invasive commensal microbiota and the abnormal activation of autoimmunity. The chronic colitis was successfully induced by 2% DSS in NOD mice. During modeling, the body weight of mice decreased, and the disease activity index score significantly increased (**Figure 3A**). Increased LPS levels in the circulation and the elevated mRNA expression of NE, MPO, PAD4 and cathelicidin antimicrobial peptide (CAMP) in the colon in DSS-treated mice (**Figures 3B, C**), manifested abnormal generation of NETs after DSS treatment. Furthermore, we observed more neutrophil infiltration and H3cit catalyzed by PAD4 existed in severely damaged areas (**Figure 3D**). In line with this, the serum levels of PAD4, NE and MPO also increased significantly in DSS-treated mice (**Figure 3E**). Moreover, the neutrophils were easier to form NETs *in vitro* after DSS administration (**Figure 3F**).

**Figure 4:**
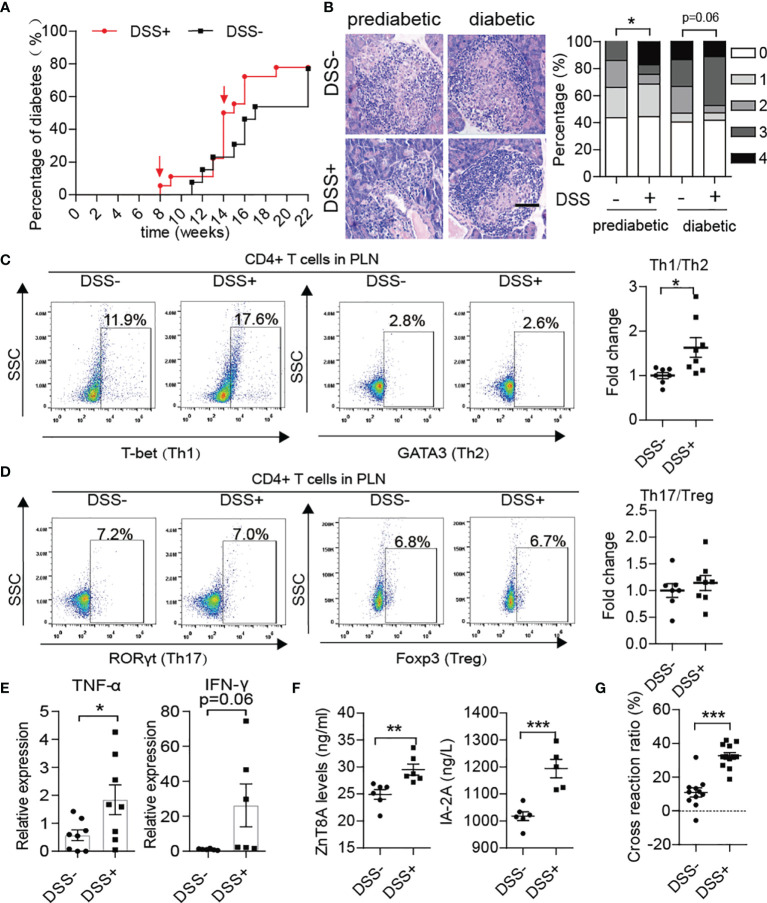
DSS treatment aggravated autoimmunity in NOD mice and accelerated the course of type 1 diabetes. **(A)** Incidence of autoimmune diabetes in control (n= 14) and DSS-induced colitis NOD mice (n= 19). **(B)** HE staining of pancreatic tissue and insulitis scores showed more mononuclear cell infiltration in DSS-colitis NOD mice than in control mice at the prediabetic and diabetic stages, respectively (n= 6). **(C, D)** Representative flow cytometry plots (left) and bar graphs with mean percentages ± SEM (right) of Th1 and Th17 within PLN of control and DSS-induced colitis NOD mice (two independent experiments; n= 3-4 mice per group). **(E)** RT-qPCR analysis of cytokine genes encoding TNF-α and IFN-γ on tissue homogenates from the pancreas of DSS-induced colitis NOD mice and the control (n= 6-8). **(F)** Detection of circulating T1D-associated autoantibodies (ZnT8-ab, IA-2A) in DSS-induced colitis NOD mice and the control (n= 6). **(G)** Cross-reaction of IAA with NET-associated autoantigens in DSS-induced colitis NOD mice (n= 12) *vs.* control mice (n= 11). The scale bars indicate 200 μm **(B)**. Data are shown as the mean ± SEM. *P < 0.05, **P < 0.01, and ***P < 0.001.

After DSS treatment, the progression of T1D disease in NOD mice was obviously accelerated. In detail, T1D onset in DSS-induced colitis NOD mice started at 8 weeks old and reached maximum morbidity at 18 weeks, which was four weeks earlier than in the control group (**Figure 4A**). In addition, a significant increase in morbidity occurred at approximately 14 weeks old, just after the fourth DSS treatment. DSS-induced colitis aggravation of islet inflammation was observed in both prediabetic and diabetic NOD mice (**Figure 4B**). Subsequently, we detected phenotypes of T helper cells in the spleen, MLN and PLN of the DSS-treated group in comparison to the control group. We found that after DSS induction, the ratio of Th1/Th2 cells in the PLN increased significantly in NOD mice (**Figure 4C**), while there was little difference in the ratio of Th17/Treg cells between the two groups (**Figure 4D**). Accordingly, the mRNA expression levels of effector inflammatory cytokines of Th1 cells, such as TNF-α and IFN-γ, were increased in the pancreas (**Figure 4E**). In addition, higher circulating levels of autoantibodies such as zinc transporter 8 protein antibody (ZnT8A) and insulinoma-associated antigen 2 antibody (IA2A) in the DSS-treated group showed greater humoral autoimmunity after gut damage (**Figure 4F**). In addition, DSS treatment doubly strengthened the cross-reaction of NET-associated autoantibodies with insulin (**Figure 4G**). Moreover, increased H3cit was discovered in the pancreas (**Supplementary Figure 3A**) in accordance with increased PAD4 levels, which could trigger the activation of autoreactive T cells (28, 29) and the generation of autoantibodies and would be another powerful explanation for the aggravation of T1D after DSS-treatment.

### PAD4 Knockout Diminished NET Formation in NOD Mice

Considering that PAD4 is essential for NET formation (30) and the generation of citrullinated protein *in vivo*, PAD4 knock out NOD mice were used to investigate whether it is possible to affect T1D development. Above all, we isolated neutrophils from PAD4-/- mice and PAD4+/+ mice to test their ability to form NETs under different stimulation conditions *in vitro* (**Supplementary Figure 4A**). The results came out that neutrophils from PAD4-/- mice indeed exhibited a lower ability to form NETs, especially when stimulated by ionomycin and microbes, which was reflected by the lower percentage of H3cit+ neutrophils and NETs (**Supplementary Figures 4B, C**), as well as the decreased serum levels of H3cit and NE in PAD4-/- mice (**Supplementary Figure 4D**). Furthermore, the ACPA also decreased in PAD4-/- mice (**Supplementary Figure 4E**). These results suggested that the activation of humoral autoimmunity to citrullinated proteins or other components of NETs were decreased to some extent by PAD4 knockout in NOD mice.

### Blocking NET Formation Improved Gut Barrier Function and Alleviated the Development of T1D

**Figure 5:**
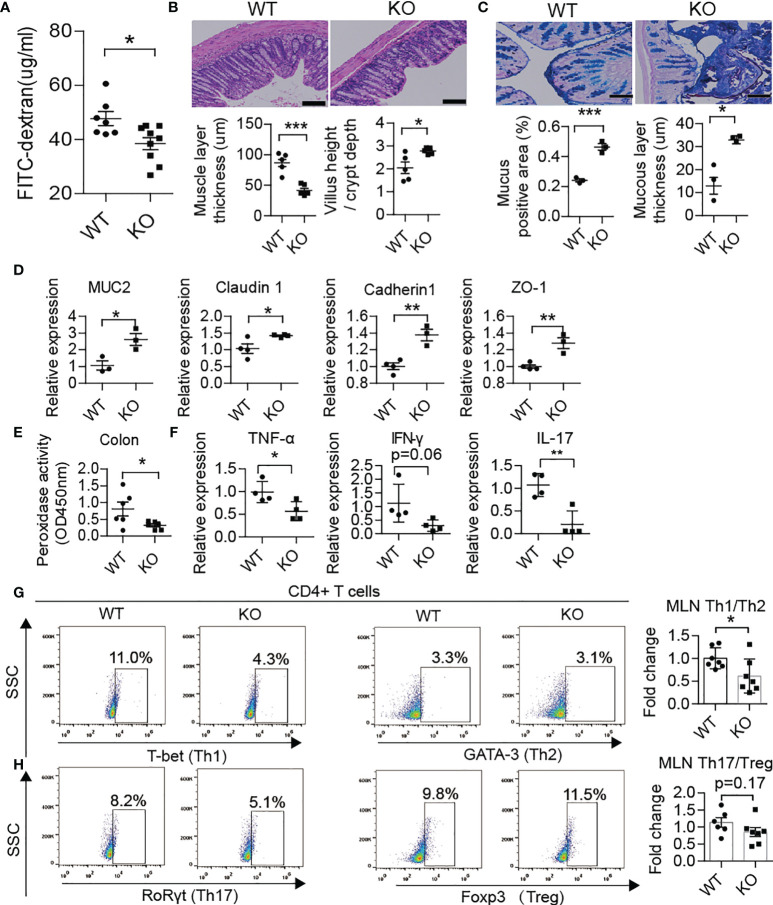
PAD4 knockout improved gut barrier function. **(A)** FITC-dextran *in vivo* permeability assay in female WT (n= 7) and KO mice (n= 9). **(B)** HE staining of colon tissue and muscle layer thickness and the ratio of the villus height/crypt depth in female WT and KO mice (n= 5). **(C)** AB-PAS staining of colon tissue and the percentage of the positive area or mucous layer thickness in female WT (n= 3) and KO mice (n= 3). **(D)** RT-qPCR analysis of genes encoding MUC2, claudin 1, cadherin1 and ZO-1 in tissue homogenates from the colon of WT (n= 4) and KO mice (n= 3). **(E)** Decreased peroxidase activity in colon homogenates of KO mice (n= 6) compared with WT mice (n= 5). **(F)** RT-qPCR analysis of genes encoding TNF-α, IFN-γ and IL-17 in tissue homogenates from the colon of WT and KO mice (n= 4). Representative flow cytometry plots (bottom) and bar graph with mean percentages ± SEM (top) of Th1 and Th2 cells and their ratio **(G)** and Th17 and Treg cells and their ratio **(H)** within the MLN of WT and KO mice (two independent experiments; n= 6-7 mice per group). PAD4-/- or PAD4+/+ mice were all after 10-12 weeks old **(A–H)**. The scale bars indicate 100 μm **(B, C)**. Data are shown as the mean ± SEM. *P < 0.05, **P < 0.01, and ***P < 0.001.

Recently, several studies have demonstrated that PAD4 inhibitor (31) or DNase I treatment could effectively achieve remission in NETs inducing colitis (32, 33). Therefore, we compared the barrier function and NET formation in the colon and enteric autoimmune T cells in PAD4-/- mice *vs.* WT mice. In PAD4 KO mice, recovery of gut barrier function was reflected at a decrease in gut permeability through FITC-dextran permeability assay (**Figure 5A**). The thinner muscle layer, higher villus height/crypt depth ratio (**Figure 5B**), higher mucus generation (**Figure 5C**), increased tight junction protein gene expression (MUC2, claudin1, cadherin1 and ZO-1) (**Figure 5D**) and claudin1 protein expression (**Supplementary Figure 3B**) in the colon also indicated recovery of the intestinal tract barrier. In addition, knockout of PAD4 resulted in lower levels of colon peroxidase activity (**Figure 5E**). Then, we focused on whether NET deficiency due to PAD4 knockout could alter the balance of T cell subsets. As expected, the ratios of Th1/Th2 (**Figure 5G**) and Th17/Treg (**Figure 5H**) cells in the MLN declined in KO mice at approximately 12 weeks of age. The mRNA expression levels of Th1 cytokines, such as TNF-α and IFN-γ, and Th17 cytokines, such as IL-17, were decreased in the colon of KO mice (**Figure 5F**).

**Figure 6:**
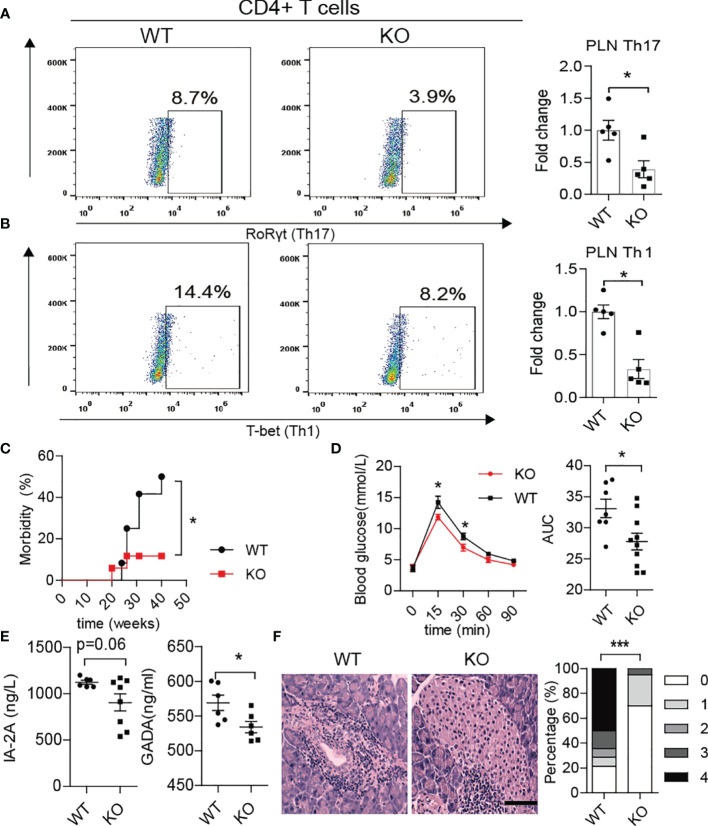
PAD4 knockout alleviated the development of T1D in NOD mice. Representative flow cytometry plots and bar graphs with mean percentages ± SEM of Th17 **(A)** and Th1 **(B)** cells within the PLN of WT and KO mice (n= 5 mice per group). **(C)** Incidence of autoimmune diabetes in WT (n= 12) and KO mice (n=15). **(D)** OGTT of nondiabetic WT (n= 7) and KO mice (n= 10). **(E)** Serum levels of IA2 and GADA autoantibodies in WT *vs.* KO mice (n= 6-8). **(F)** HE staining of pancreatic tissue and insulitis score showing more mononuclear cells infiltration in WT mice than in KO mice (n= 3). PAD4-/- or PAD4+/+ mice were all after 12 weeks old **(A–F)**. The scale bars indicate 200 μm **(F)**. Data are shown as the mean ± SEM. *P < 0.05 and ***P < 0.001.

Interestingly, we also found fewer Th1/Th17 cells in the PLN of KO mice at approximately 10-12 weeks of age (**Figures 6A, B**). The KO mice displayed better glucose tolerance and lower morbidity than WT mice (**Figures 6C, D**). More importantly, the serum levels of autoantibodies such as glutamic acid decarboxylase antibody (GADA) or IA2A decreased in KO mice (**Figure 6E**). In line with this, HE staining showed less inflammation in the pancreas of KO mice, which confirmed the alleviation of autoimmunity after PAD4 knockout (**Figure 6F**). To further verify the hypothesis that NETs mediate the loss of the gut barrier and activation of autoimmune T cells, we orally administered 2.5% DSS to both KO and WT mice for 7 days to destroy the intestinal barrier function. The results showed that the expression of IFN-γ in the pancreas was significantly higher in WT mice but not KO mice (**Supplementary Figure 5A**) after colitis induction. In addition, the migration capacity of enteric lymphocytes in KO mice after DSS treatment to MIN6 cells *in vitro* was weaker than that in WT mice with colitis (**Supplementary Figure 5B**). Most importantly, we observed a significant increase in enteric CD4+ T cells (integrin α4β7 positive) (**Supplementary Figure 5C**), especially Th17 and Th1 cells (**Supplementary Figures 5D, E**) in the PLNs of WT mice but not KO mice with colitis. Meanwhile, the expression of integrin α4/β7 showed the same differences in the pancreas (**Supplementary Figure 5F**). Overall, our data demonstrated that NET deficiency prevents autoimmune T cells from traveling from GALT to the PLN to cause pancreatic beta cells destruction.

## Discussion

Accumulating evidence has demonstrated that gut barrier dysfunction and commensal microbial disorders could be critical pathogenic factors triggering T1D (34). However, knowledge of how gut leakage and invasive gut microbes in T1D patients or animal models promote beta cells autoimmunity is still limited. In the current study, we have demonstrated that excessive NET formation caused by gut leakage may be one critical factor link between innate and adaptive immune responses.

In general, susceptible individuals, chronic enteric infections first activate the innate immune response and subsequently trigger autoimmunity (35). A previous clinical observation has shown that LPS exposure arising primarily from Bacteroides precludes early immune education and contributes to the development of T1D (36). Our previous clinical study has demonstrated that circulating LPS concentration is elevated in new-onset T1D patients, while the increased internal LPS levels are relate to the changes of bacteria from Deferribacteres or Proteobacteria in NOD mice after the development of diabetes, which usually appears over 12 weeks of age. Similar to the previous study, neutrophils in NOD mice in the current study were also liable to form NETs and resulted in abnormal oscillation of the circulating levels of cf-DNA, H3cit and PAD4 at the prediabetic or diabetic stage compared with BALB/c mice of the same age. Importantly, a strong positive correlation between LPS and these biomarkers of NETs was found, which suggested that invasive gram-negative bacteria were potent in triggering NET formation *in vivo* after gut barrier disruption. Furthermore, we found that neutrophil infiltration and NET formation was increased in the leaky gut of NOD mice but not in BALB/c mice.

DNA and cellular contents released by NETs can be taken up by DCs (37) and can effectively induce the polarization of naïve T cells to Th1 and Th17 cells (38, 39). In the current study, we demonstrated for the first time that the population of memory inflammatory T cells were increased in mononuclear cells after coculturing with autologous NETs in NOD mice. Furthermore, after stimulation with NETs, enteric lymphocyte cells from MLNs exhibited a stronger migration ability to mouse insulinoma cells, which suggested the role of NETs in promoting self-islet reactive immune cell generation in GALT of NOD mice. In addition, increased NET-associated autoantibodies and the cross-reaction of IAA with NET contents in NOD mice revealed that NETs could trigger autoimmune processes in this T1D animal model.

**Figure 7:**
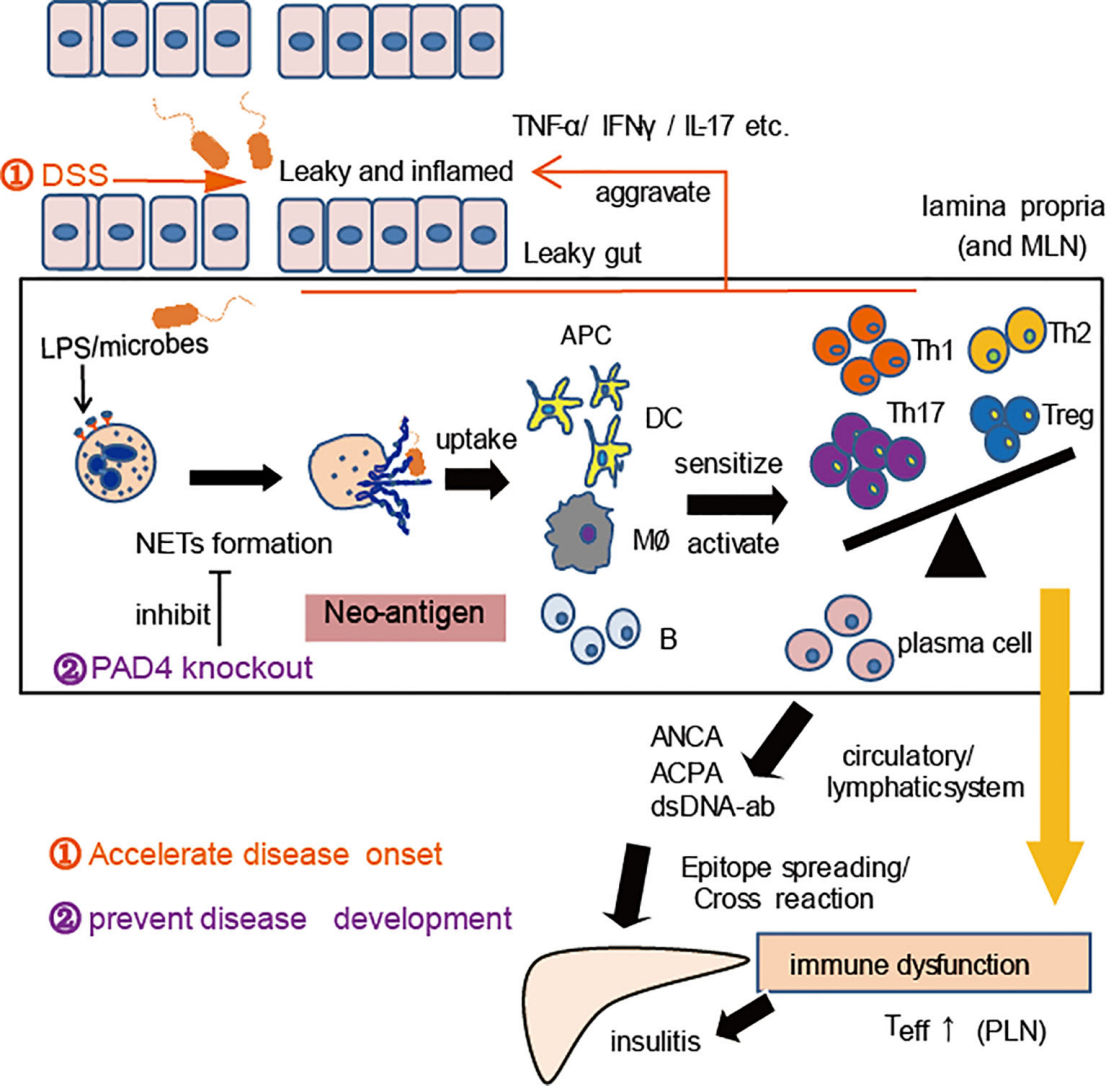
A proposed mechanism for NET to mediate gut leakage, triggering T1D by activating the autoimmune response in NOD mice. Abnormal NET formation caused by gut barrier dysfunction and abnormal microbiota could trigger the activation of autoimmune responses and ultimately induce islet destruction. DSS treatment worsens the situation and accelerates disease onset, while PAD4 deficiency leading to a decrease of NET formation results in restoring gut barrier integrity and showing the potential of T1D prevention.

The pathogenic role of NETs in DSS-induced colitis has been widely proven (32, 40), and PAD4 plays critical role in this process (41). In the current study, our induction of chronic colitis in NOD mice also proved that gut leakage occurred together with the increased NET formation *in vivo*. Thereby, we observed aggravation of autoimmunity or insulitis and a stronger cross-reaction of IAA with NET contents in DSS-induced colitis mice, as well as the acceleration of T1D development, which suggested that the higher the exposure of NETs *in vivo*, the more serious the self-damage. According to the recent reports, PAD4-deficient mice may hardly generate NETs *in vivo* and barely affect other functions of neutrophils (30, 42, 43). Moreover, compared to healthy controls, neutrophils from T1D patients tended to form NETs after ionomycin stimulation (24), which indicated that PAD4-dependent NETs might contribute to the disease. When we knocked out PAD4 in NOD mice, neutrophils’ ability to generate NETs was found to decrease significantly. Interestingly, the recovery of gut barrier function and a reduction in autoimmune T cells were also found in our PAD4-deficient mice, in terms of fewer autoimmune Th1/Th17 cells in the PLN or autoantibodies and milder insulitis, which resulted in lower disease incidence and better glucose tolerance. The heterodimeric integrin α4β7 expressed on T cells, specifies the recruitment of T cells to the intestinal mucosa upon its interaction with its ligand (26). In this study, a prominent increase in integrin α4β7-positive T cells in PLNs was discovered in WT but not in KO mice after DSS administration, in line with the elevated expression of integrin α4β7 in the pancreas. The data indicated that the improvement of gut barrier function and suppression of gut inflammation through affecting PAD4-dependent NET formation could control T1D development, which enriched the previous gut-affecting pancreas theory. Taking together, we have represented a schematic diagram to explain this mechanism in **Figure 7**.

In conclusion, the current study provides evidence that PAD4-dependent NET formation plays an essential mediating role in the gut-pancreas axis by modulating the migration of enteric autoimmune T cells and activating autoantibodies, suggesting that clearing abnormal NETs in the gut with nuclease or blocking NET formation with PAD4 inhibitors may be a new therapy approach for T1D prevention and treatment.

## Data Availability Statement

The datasets presented in this study can be found in online repositories. The names of the repository/repositories and accession number(s) can be found below: NCBI [accession: PRJNA766442].

## Ethics Statement

The animal study was reviewed and approved by Animal Laboratory Center of China Pharmaceutical University.

## Author Contributions

JW conceived the study. QY and YW designed and performed experiments, analyzed experimental data, and prepared the manuscript. YS contributed to the cells experiment. JW, YL, CL, and FH supervised the research. JW, HG, and YS interpreted the data and revised the manuscript. QY and YS contributed equally to this work. All authors contributed to the article and approved the submitted version.

## Funding

This work was supported by National Natural Science Foundation of China (Grand No. 81673340, 81973224), the Priority Academic Program Development of Jiangsu Higher Education Institutions (PAPD) and “Double First-Class” University project (Grand Nos. CPU2018GF/GY16, CPU2018GF/GY17).

## Conflict of Interest

The authors declare that the research was conducted in the absence of any commercial or financial relationships that could be construed as a potential conflict of interest.

## Publisher’s Note

All claims expressed in this article are solely those of the authors and do not necessarily represent those of their affiliated organizations, or those of the publisher, the editors and the reviewers. Any product that may be evaluated in this article, or claim that may be made by its manufacturer, is not guaranteed or endorsed by the publisher.

## Supplementary Material

Supplementary Figure 1 Commensal microbial disorders in NOD mice after 12 weeks of age. **(A)** Phylum-level phylogenetic classification of 16S rRNA frequencies in the fecal pellets of NOD/LtJ mice at 4, 8 and 12 weeks old and at the disease onset stage (n=5-6). Relative abundance of *Deferribacteres* and *Proteobacteria* **(B)**, microbial principal coordinate analysis **(C)** and 16S sequencing comparisons **(D)** in NOD/LtJ mice before 12 weeks of age *vs.* after 12 weeks of age. The bacterial families shown represent those found to be significantly different in the pairwise comparison. **(E)** Relative abundance of *Desulfovibrionaceae* and *Deferribacteraceae* in NOD/LtJ mice at 4 and 12 weeks old and at the disease onset stage. **P* < 0.05, ***P* < 0.01, and ****P* < 0.001.

Supplementary Figure 2 Gut barrier dysfunction and autoimmune disorders in NOD mice compared with BALB/c mice. **(A)** Hematoxylin and eosin staining of colon tissue in female BALB/c and NOD mice. **(B)** Muscle layer thickness, gut damage score and the ratio of villus height/crypt depth in female BALB/c and NOD mice (n=3). **(C)** RT-qPCR analysis of cytokine genes encoding TNF-α, interleukin-1β (IL-1β) and IL-17A in tissue homogenates from the colons of NOD and BALB/c mice (n=5-7). Flow cytometry shows the bar graph with mean percentages ± SEM of Th17 **(D)**, Th1 **(E)** and Treg **(F)** cells within the MLNs of NOD and BALB/c mice (two independent experiments, n=3 each group every time). BALB/c and NOD mice were all after 12 weeks old **(A–C)**. The scale bars indicate 100 μm **(A)**. Data are shown as the mean ± SEM. **P* < 0.05 and ****P* < 0.001.

Supplementary Figure 3 Western blotting analysis of h3cit in pancreas and Claudin1 in colons. **(A)** Western blotting analysis of h3cit from pancreas homogenates in DSS-colitis NOD mice (n=8) and the control (n=6). **(B)** Western blotting analysis of claudin 1 from colon tissue of WT and KO mice (n=3). Data are shown as the mean ± SEM. **P* < 0.05.

Supplementary Figure 4 PAD4 knockout diminished neutrophil NET formation. **(A)** Detection of neutrophils from PAD4^-/-^ or PAD4^+/+^ mice (n= 3), forming NETs under different stimulation conditions [DMSO, ionomycin (10 μM), PMA (8 μM), fecal microbes (2*10^7^)] *in vitro*. The percentage of cells that were hypercitrullinated at histone H3 **(B)** and produced NETs **(C)** in neutrophils was calculated (three quantifications for each mouse). **(D)** Lower serum levels of NET biomarkers were detected in KO mice than in WT mice (n= 6-9). **(E)** Detection of CCPA in WT and KO mice (n=6). PAD4^-/-^ or PAD4^+/+^ mice were all after 10-12 weeks old **(A–E)**. The scale bars indicate 100 μm **(A)**. Data are shown as the mean ± SEM. **P* < 0.05 and ***P* < 0.01.

Supplementary Figure 5 PAD4 deficiency diminished enteric lymphocyte T cells traveling to the pancreas. **(A)** RT-qPCR analysis of cytokine genes encoding IFN-γ in tissue homogenates from the pancreas of WT and KO mice with or without DSS treatment (n=3-4). **(B)** The Transwell system shows the migration capacity of lymphocyte cells from MLNs in WT and KO mice with or without DSS treatment (n=3). The percentage and number of enteric CD4^+^ T **(C)**, Th17 **(D)** and Th1 **(E)** cells in PLNs from WT and KO mice with or without DSS treatment (n=3). **(F)** RT-qPCR analysis of integrin α4/β7 in tissue homogenates of pancreas from WT and KO mice with or without DSS treatment (n=3-4). PAD4^-/-^ or PAD4^+/+^ mice were all after 10-12 weeks old **(A–F)**. Data are shown as the mean ± SEM. **P* < 0.05.
