# Supplementary material for: Neutrophil Extracellular Traps Caused by Gut Leakage Trigger the Autoimmune Response in Nonobese Diabetic Mice
Source: Front Immunol. 2022 Jan 17;12:711423. doi: 10.3389/fimmu.2021.711423 (PMC8801438; doi:10.3389/fimmu.2021.711423)
Supplement: Supplementary Figure 1 — Commensal microbial disorders in NOD mice after 12 weeks of age. (A) Phylum-level phylogenetic classification of 16S rRNA frequencies in the fecal pellets of NOD/LtJ mice at 4, 8 and 12 weeks old and at the disease onset stage (n=5-6). Relative abundance of Deferribacteres and Proteobacteria (B), microbial principal coordinate analysis (C) and 16S sequencing comparisons (D) in NOD/LtJ mice before 12 weeks of age vs. after 12 weeks of age. The bacterial families shown represent those found to be significantly different in the pairwise comparison. (E) Relative abundance of Desulfovibrionaceae and Deferribacteraceae in NOD/LtJ mice at 4 and 12 weeks old and at the disease onset stage. *P < 0.05, **P < 0.01, and ***P < 0.001. [file Image_1.pdf]

Supplemental Figure 1

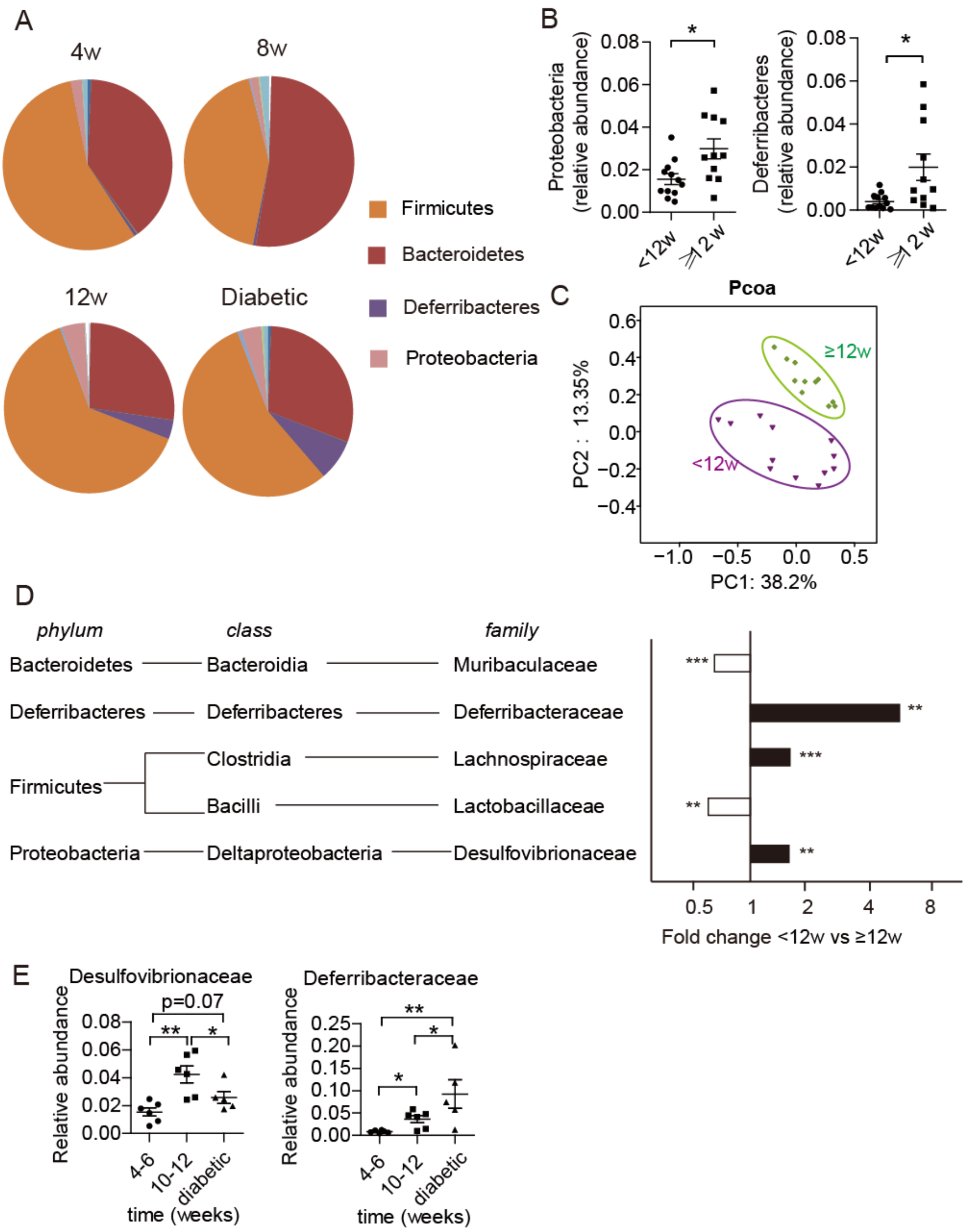

Supplemental Figure 2

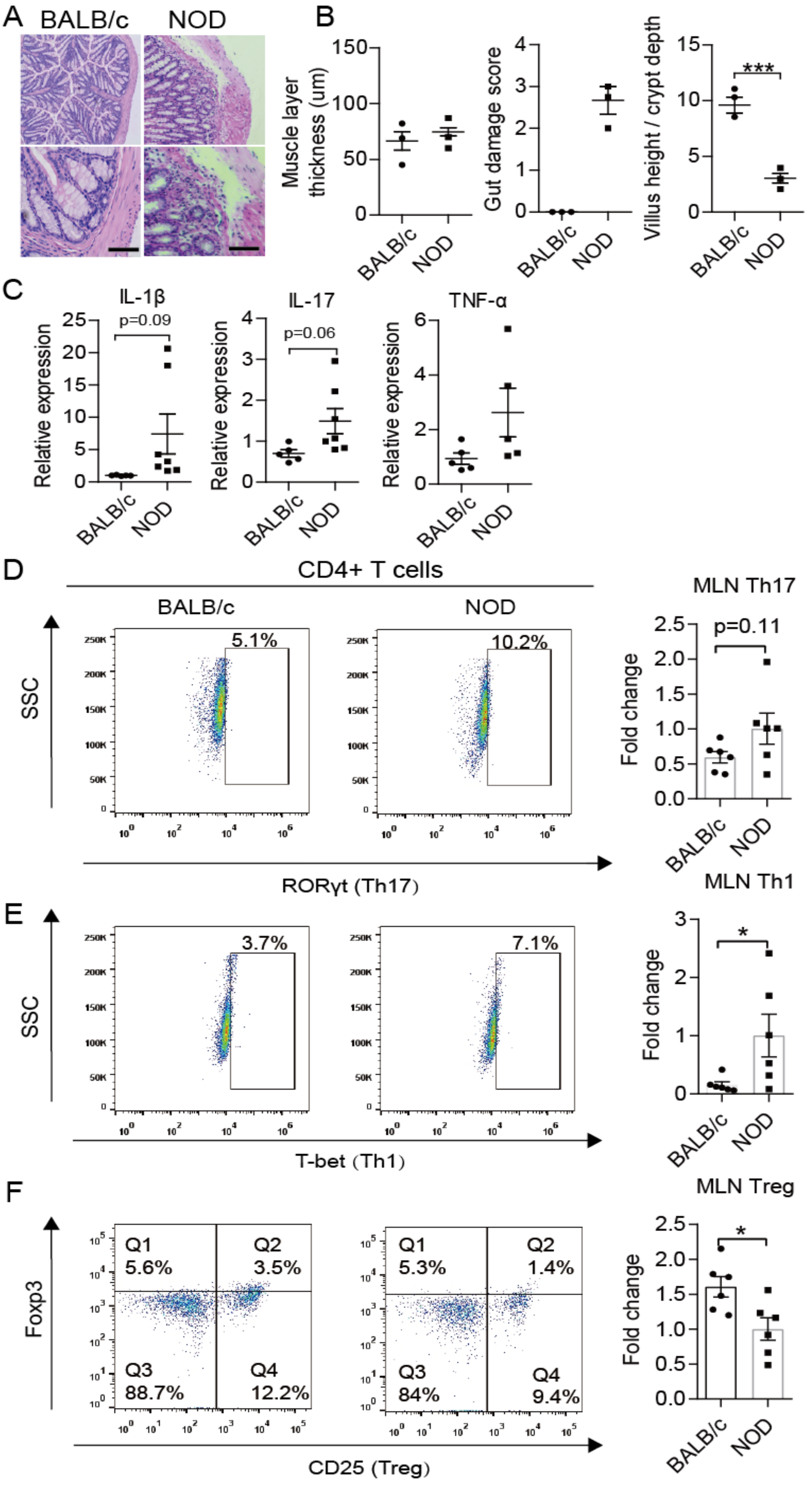

Supplemental Figure 3

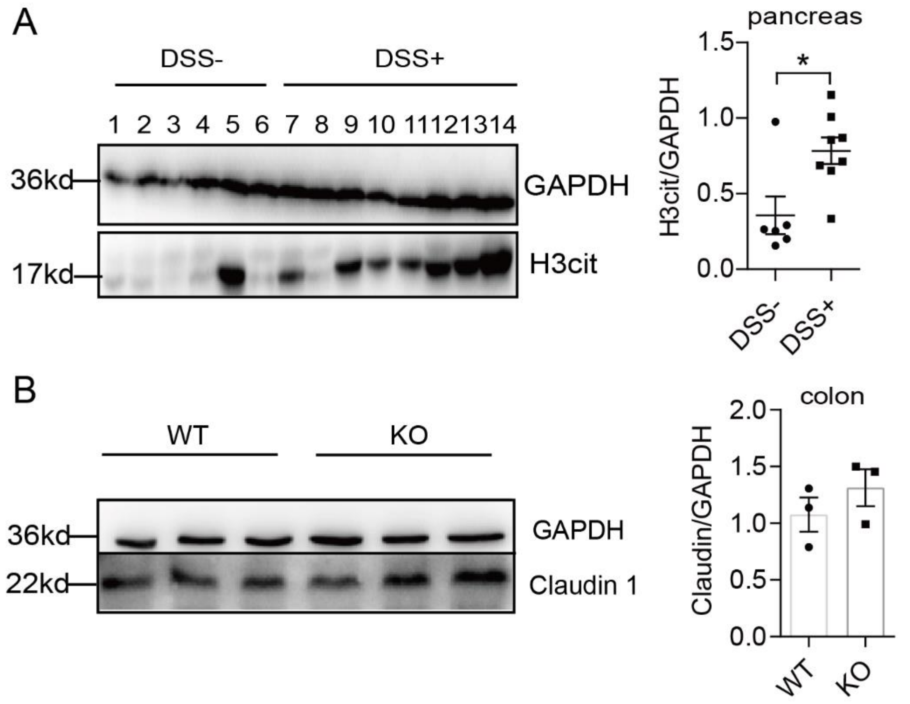

Supplemental Figure 4

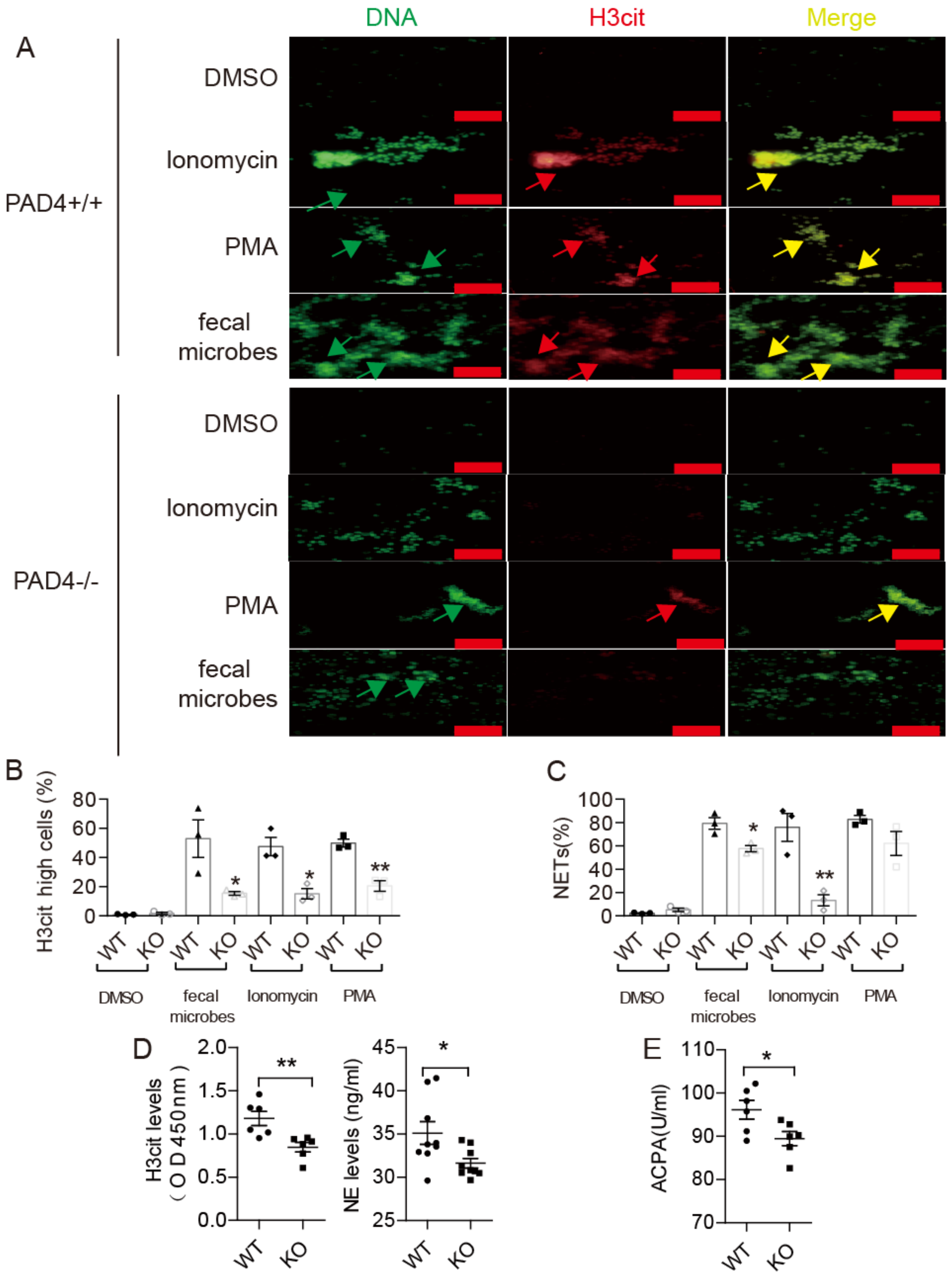

Supplemental Figure 5

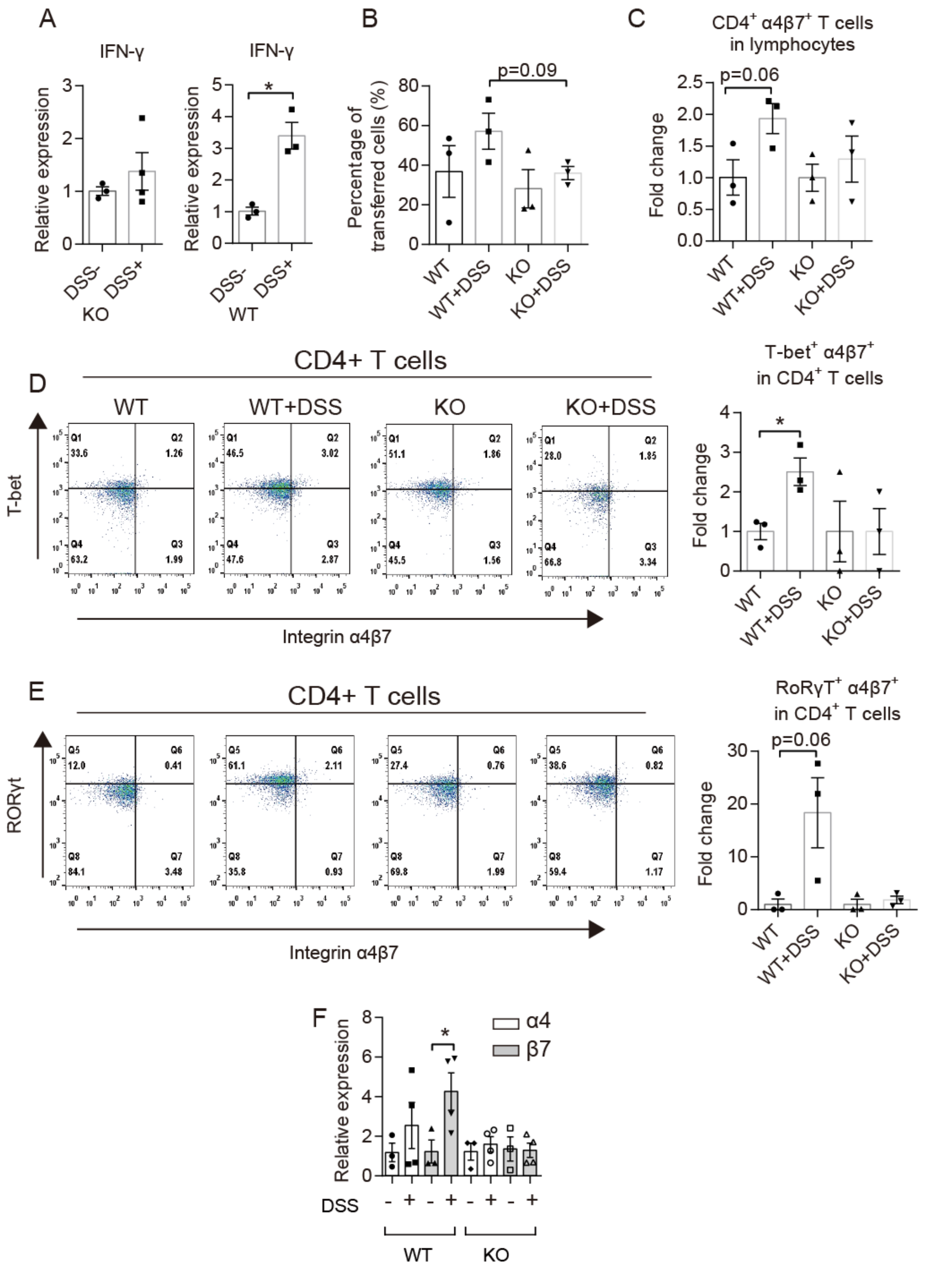

Supplemental Figure 1: Commensal microbial disorders in NOD mice after 12 weeks of age. (A) Phylum-level phylogenetic classification of 16S rRNA frequencies in the fecal pellets of NOD/LtJ mice at 4, 8 and 12 weeks old and at the disease onset stage (n=5-6). Relative abundance of *Deferribacteres* and *Proteobacteria* (B), microbial principal coordinate analysis (C) and 16S sequencing comparisons (D) in NOD/LtJ mice before 12 weeks of age vs. after 12 weeks of age. The bacterial families shown represent those found to be significantly different in the pairwise comparison. (E) Relative abundance of *Desulfovibrionaceae* and *Deferribacteraceae* in NOD/LtJ mice at 4 and 12 weeks old and at the disease onset stage. *$P < 0.05$, **$P < 0.01$, and ***$P < 0.001$.

Supplemental Figure 2: Gut barrier dysfunction and autoimmune disorders in NOD mice compared with BALB/c mice. (A) Hematoxylin and eosin staining of colon tissue in female BALB/c and NOD mice. (B) Muscle layer thickness, gut damage score and the ratio of villus height/crypt depth in female BALB/c and NOD mice (n=3). (C) RT-qPCR analysis of cytokine genes encoding TNF-α, interleukin-1β (IL-1β) and IL-17A in tissue homogenates from the colons of NOD and BALB/c mice (n=5-7). Flow cytometry shows the bar graph with mean percentages ± SEM of Th17 (D), Th1 (E) and Treg (F) cells within the MLNs of NOD and BALB/c mice (two independent experiments, n=3 each group every time). BALB/c and NOD mice were all after 12 weeks old (A-C). The scale bars indicate 100 μm (A). Data are shown as the mean ± SEM. *$P < 0.05$ and ***$P < 0.001$.

Supplemental Figure 3: Western blotting analysis of h3cit in pancreas and Claudin1 in colons.

(A) Western blotting analysis of h3cit from pancreas homogenates in DSS-colitis NOD mice (n=8) and the control (n=6). (B) Western blotting analysis of claudin 1 from colon tissue of WT and KO mice (n=3). Data are shown as the mean ± SEM. *$P < 0.05$.

Supplemental Figure 4: PAD4 knockout diminished neutrophil NET formation. (A) Detection of neutrophils from $PAD4^{-/-}$ or $PAD4^{+/+}$ mice (n= 3), forming NETs under different stimulation conditions [DMSO, ionomycin (10 μM), PMA (8 μM), fecal

microbes ($2*10^7$)] *in vitro*. The percentage of cells that were hypercitrullinated at histone H3 (B) and produced NETs (C) in neutrophils was calculated (three quantifications for each mouse). (D) Lower serum levels of NET biomarkers were detected in KO mice than in WT mice (n= 6-9). (E) Detection of CCPA in WT and KO mice (n=6). $PAD4^{-/-}$ or $PAD4^{+/+}$ mice were all after 10-12 weeks old (A-E). The scale bars indicate 100 μm (A). Data are shown as the mean ± SEM. *P < 0.05 and **P < 0.01.

Supplemental Figure 5: PAD4 deficiency diminished enteric lymphocyte T cells traveling to the pancreas. (A) RT-qPCR analysis of cytokine genes encoding IFN-γ in tissue homogenates from the pancreas of WT and KO mice with or without DSS treatment (n=3-4). (B) The Transwell system shows the migration capacity of lymphocyte cells from MLNs in WT and KO mice with or without DSS treatment (n=3). The percentage and number of enteric $CD4^+$ T (C), Th17 (D) and Th1 (E) cells in PLNs from WT and KO mice with or without DSS treatment (n=3). (F) RT-qPCR analysis of integrin α4/β7 in tissue homogenates of pancreas from WT and KO mice with or without DSS treatment (n=3-4). $PAD4^{-/-}$ or $PAD4^{+/+}$ mice were all after 10-12 weeks old (A-F). Data are shown as the mean ± SEM. *P < 0.05.
