# Supplementary material for: Neutrophil Extracellular Traps Caused by Gut Leakage Trigger the Autoimmune Response in Nonobese Diabetic Mice
Source: Front Immunol. 2022 Jan 17;12:711423. doi: 10.3389/fimmu.2021.711423 (PMC8801438; doi:10.3389/fimmu.2021.711423)
Supplement: Supplementary file 2 [file Table_1.pdf]

Supplemental Table 1.

Primer sequence

Gene	Primers
Cadherin 1	FP: CAGTTCCGAGGTCTACACCTT; RP: TGAATCGGGAGTCTTCCGAAAA
CAMP	FP: GCTGTGGCGGTCACTATCAC; RP: TGTCTAGGGACTGCTGGTTGA
Claudin 1	FP: AGCACCGGGCAGATACAGT; RP: GCCAATTACCATCAAGGCTCG
Cldn1	FP: TGCCCCAGTGGAAGATTTACT; RP: CTTTGCGAAACGCAGGACT
GAPDH	FP: AGGTCGGTGTGAACGGATTTG; RP: TGTAGACCATGTAGTTGAGGTCA
IL-1β	FP: GAAATGCCACCTTTTGACAGTG; RP: TGGATGCTCTCATCAGGACAG
IL-17	FP: TCAGCGTGTCCAAACACTGAG; RP: CGCCAAGGGAGTTAAAGACTT
IFN-γ	FP: GCCACGGCACAGTCATTGA; RP: TGCTGATGGCCTGATTGTCTT
Intergrin α4	FP: CCTCAGTGGTCAATCCTGGG; RP: CACAAGTCACGATAGAGCCATT
Intergrin β7	FP: ACCTGAGCTACTCAATGAAGGA; RP: CACCGTTTTGTCCACGAAGG
MPO	FP: AGTTGTGCTGAGCTGTATGGA; RP: CGGCTGCTTGAAGTAAAACAGG
MUC2	FP: AGGGCTCGGAACTCCAGAAA; RP: CCAGGGAATCGGTAGACATCG
NE	FP: AGCAGTCCATTGTGTGAACGG; RP: CACAGCCTCCTCGGATGAAG
Occludin	FP: TGAAAGTCCACCTCCTTACAGA; RP: CCGGATAAAAAGAGTACGCTGG
PAD4	FP: GACCACAACAGTTCTCGTATTGC; RP: CGGGTTAGACTTGTCCAGCAG
TNF-α	FP: CTGAACTTCGGGGTGATCGG; RP: GGCTTGTCACTCGAATTTTGAGA
V4-V5 515F-907R	FP: GTGCCAGCMGCCGCGG; RP: CCGTCAATTCMTTTRAGT
ZO-1	FP: GGGCCATCTCAACTCCTGTA; RP: AGAAGGGCTGACGGGTAAAT

FP: forward primer; RP: reverse primer
